# Hydroxychloroquine and QT prolongation: reassuring data in approved indications

**DOI:** 10.1093/rap/rkaa044

**Published:** 2020-08-21

**Authors:** Anil Pareek, Tarun S Sharma, Ravi Tejraj Mehta

**Affiliations:** Medical Affairs, Ipca Laboratories Limited, Mumbai, India; Division of Rheumatology, Allegheny Health Network, Pittsburgh, PA, USA; Medical Affairs, Ipca Laboratories Limited, Mumbai, India

**Key message**

- HCQ-induced QT prolongation does not pose a major safety issue at the recommended doses in approved indications in rheumatology.

Sir, Severe acute respiratory syndrome coronavirus 2 (SARS-CoV-2) has infected almost 8 million individuals globally as of 15 June 2020 and is still spreading at an alarming rate. An efficient approach to drug discovery in this scenario would be to identify drugs that could be repurposed with minimal further testing, and HCQ is one such agent being evaluated extensively. Based on the limited observational studies published to date, concerns have been raised on the cardiovascular safety of HCQ, particularly in combination with azithromycin [1]. This has also given rise to concern among physicians and patients with rheumatic diseases who have been using HCQ for several decades. It is important to understand the differences in these two distinct clinical settings where HCQ is being used. 

Coronavirus disease (COVID-19) is a pro-arrhythmogenic state created by direct viral myocardial damage, hypoxia, hypotension, enhanced inflammatory status, electrolyte abnormalities, concomitant QT-prolonging medications and underlying cardiovascular disease, with the risk being higher in patients with severe COVID-19. In one of the earliest reports on clinical characteristics, arrhythmias were reported in 17% of hospitalized patients and in 44% of patients admitted to the intensive care unit with COVID-19 [2]. In most of the clinical studies published to date, HCQ has been administered to hospitalized COVID-19 patients who were critically ill, requiring intensive care and/or taking at least two QTc-prolonging medications, such as azithromycin and oseltamivir. 

HCQ has been in clinical use for >60 years, with an excellent risk:benefit ratio in approved indications, including RA and lupus. Safety reporting of an established molecule, such as HCQ, should be database driven rather than based on observational data. Apart from a few isolated case reports, the incidence of HCQ-associated QT prolongation at recommended doses appears to be rare. The Electronic Medicines Compendium regulated by UK Medicines and Healthcare Products Regulatory Agency and the European Medicines Agency reports the frequency rating of side effects as follows: very common, ≥10%; common, ≥1 and <10%; uncommon, ≥0.1 and <1%; rare, ≥0.01 and <0.1%; very rare, <0.01%; and not known (frequency cannot be estimated from available data). The cardiac adverse effects of HCQ, including cardiomyopathy and conduction disorders, feature in the category of not known [3]. Furthermore, although drug-induced QT prolongation is a surrogate indicator for an increased risk of drug-associated torsades de pointes, the risk of torsades de pointes or arrhythmic death is not a linear function of basal QT duration or the extent of drug-induced prolongation of QTc [4]. In conjunction with this, the ability of HCQ to prolong the QT interval does not appear to be associated with a substantial risk of sudden cardiac death and torsades de pointes. 

In a recent analysis from the University of Florida, >13 million reports over the last 50 years (from 1969 to 3rd Quarter of 2019) from the U.S. Food and Drug Administration’s Adverse Event Reporting System were scrutinized [5]. The U.S. Food and Drug Administration’s Adverse Event Reporting System reports include adverse drug events reported via mandatory reporting by pharmaceutical companies in addition to voluntary reporting by consumers and have the ability to capture rare safety events, such as torsades de pointes/QT prolongation. HCQ was not associated with a safety signal related to torsades de pointes/QT prolongation or death when used alone in these approved indications, whereas azithromycin with or without HCQ was associated with QT prolongation. In an analysis of ECGs in 85 patients with connective tissue diseases treated with HCQ for >1 year, the corrected QT (QTc) interval was not different from normal values, and no atrioventricular block was observed [6]. ECG monitoring is not part of standard clinical practice while using HCQ as monotherapy in rheumatology. Nonetheless, a baseline ECG is a simple, useful and inexpensive screening clinical tool in potentially high-risk individuals, such as those with structural heart diseases, especially ventricular hypertrophy or left ventricular dysfunction, a history of ventricular arrhythmia or syncope.

Together with the comforting safety data for HCQ in rheumatic conditions, metabolic and cardiovascular benefits have been associated with its use in these approved indications [7]. Wide emphasis on the rare cardiac effects might induce unnecessary anxiety in patients taking HCQ at recommended doses for rheumatic conditions, in which good-quality evidence suggests reduced risk of cardiovascular disease [8, 9]. Conversely, there is a known risk of flare related to HCQ withdrawal in patients with these approved indications. In the landmark study by the Canadian Hydroxychloroquine Study Group, withdrawal of HCQ in patients with stable lupus showed a 2.5 times higher risk of flare.

Thus, it is crucial that prescribers and patients using HCQ for approved indications be reassured about its continued usage. Global rheumatology societies, including the British Society for Rheumatology, ACR and Asia Pacific League of Associations for Rheumatology have opined that existing patients with rheumatic diseases can continue HCQ (and SSZ) if they are infected with coronavirus, whereas other conventional DMARD and biologic therapy must be stopped temporarily. Together with the clinical and pharmacovigilance data, such position statements from rheumatology societies must reassure the physicians and patients about the safety of this time-tested and useful agent in rheumatology.

*Funding*: No specific funding was received from any funding bodies in the public, commercial or not-for-profit sectors to carry out the work described in this manuscript.

*Disclosure statement*: A.P. and R.T.M. are employees of Ipca Laboratories Limited and are involved in clinical research on HCQ. The remaining author has declared no conflicts of interest.
